# The Effect and Mechanism of Cultural Capital on Chinese Residents’ Participation in Physical Activities

**DOI:** 10.3389/fpsyg.2022.848530

**Published:** 2022-04-21

**Authors:** Huitao Ren, Wei Liu

**Affiliations:** ^1^Sport and Health College, Wenzhou University, Wenzhou, China; ^2^Physical Education College, Quanzhou Normal University, Quanzhou, China; ^3^Key Research Base of Humanities and Social Sciences in Colleges and Universities in Anhui Province–Quality Education Research Center for College Students of Anhui Xinhua University, Hefei, China; ^4^Department of Physical Education, Anhui Vocational and Technical College of Sports, Hefei, China

**Keywords:** residents, health self-assessment, income, participation, physical activity, cultural capital

## Abstract

**Background:**

Using Bourdieu’s cultural capital theory, this paper discusses the inequality of Chinese urban residents’ participation in physical activities caused by cultural capital and explores the relationship and role of residents’ income and self-rated health in cultural capital and physical activity participation.

**Methods:**

Using Chinese social survey data (2017), the proposed assumptions were tested and analyzed by using a linear regression model.

**Results:**

Cultural capital can promote the participation of Chinese urban residents in physical activities, and personal income and health self-assessment play an intermediary role in promoting residents’ participation in physical activities.

**Conclusion:**

Promoting Chinese residents’ participation in physical activities can be realized not only through traditional publicity and sports venue construction but also by increasing residents’ educational level, art appreciation level and health awareness.

## Introduction

Cultural capital generally refers to any tangible or intangible asset related to culture and cultural activities. Cultural capital can be divided into three basic forms: physical form, objective form and institutional form (Bourdieu, 1986). It can be seen that cultural capital is a form of capital that expresses the cultural advantages or disadvantages of actors. Physical activity is not only an important option in residents’ daily lives but also an important way to improve residents’ physical and mental health and life happiness. Therefore, the study of urban residents’ participation in physical activities is an important part of China’s 2030 health strategy and national fitness plan. Compared with other daily life behaviors of residents, the participation of physical activities is related to residents’ income, education, leisure time, interest and other factors, which makes it particularly important to deeply study the influencing factors of physical activities. The problem of physical activity participation is not only a person’s choice but also involves factors such as the family environment and social environment. Therefore, early research on the influence effect and mechanism of Chinese residents’ participation in physical activities basically focused on the characteristics of residents’ individual demographic characteristics, economic factors and so on. However, it soon turned to the discussion of structural elements such as social stratification and class differences. Recently, it has focused on discussion from the perspectives of social capital and reverse effects. However, in past research, there has been little literature exploring the influence effect and mechanism of cultural capital on residents’ participation in physical activities.

“Cultural capital” is a sociological concept extended by Bourdieu after summarizing Marx’s concept of capital. Cultural capital is a form of capital that expresses the cultural advantages or disadvantages of actors. It can be transformed into economic capital under specific conditions. The whole transformation process is institutionalized in the form of educational qualifications (Li and Liu, 2021). He also believes that modern politics can no longer solve problems only by political means, economic problems cannot rely on the market to solve all problems, and the concept of cultural capital can eliminate common sense concepts and human capital assumptions. In particular, he believes that the solution to social problems largely depends on the distribution of cultural capital between social classes and class groups. Bourdieu divided cultural capital into three basic forms: concrete cultural capital, objective cultural capital and systematic culture capital. Among them, specific cultural capital generally refers to the knowledge, literacy, taste, and other cultural products obtained through family and school education. Objective cultural capital specifically refers to material and cultural wealth such as books, antiques, and tools, which is a kind of cultural capital that can be directly transmitted. Systematic culture capital refers to the capital form in which the knowledge and skills of actors are confirmed by examination and institutionalized by diploma. The differences in the acquisition of the three forms of cultural capital among individual residents lead to the level of individual cultural capital accumulation and then shape the differences in knowledge level, health awareness and sports cognition among residents, affecting the choice differences of residents in participating in physical activities. Bourdieu (1979), when discussing the specific concept of cultural capital, found and proved that physical activity is related to whether a person can obtain higher cultural capital (Bourdieu and Passeron, 1979). Specifically, it may be education level, ability to understand culture and compliance with cultural norms (Warde, 2006). To further study the role of cultural capital in shaping social structure, Bourdieu summarized cultural capital into three dimensions. Institutional cultural capital is the first dimension, which is reflected in the individual’s educational acquisition, such as degree certificates and various qualification certificates. It is a sign that the individual’s knowledge, skills and abilities are recognized by society. These recognized “cultural signs” institutionalize and symbolize the individual’s cultural acquisition, so they have received higher-level education. Obtaining more qualification certificates means that the cultural capital of the individual system is also higher. Embodied cultural capital is the second dimension, which is embodied in lasting temperament and physical and mental quality, including values, preferences, codes of conduct and operating skills. This content is gradually accumulated through individual cultural attitudes and social practice, such as participating in concerts, museums, movies, art performances and other cultural activities. Objective cultural capital is the third dimension, which is embodied in materialized cultural products, such as art works and books owned by individuals. Having more materialized cultural products means that individuals have better conditions to absorb cultural nutrients and accumulate cultural knowledge, and their objective cultural capital level is also higher. The difference in individual accumulation in the three dimensions of cultural capital affects the overall level of self-cultural capital acquisition, and different levels of cultural capital will shape individual differentiated physical and mental characteristics and may also make individuals form differentiated social behavior, which will affect individual concepts, education, income and even health (Dumais, 2009).

Through the combination of the concept of cultural capital, we know that the inequality of the results of cultural capital may lead to differences in residents’ choices in participating in physical activities. However, the existing research results have not yet involved the mechanism of cultural capital and physical activities. This paper innovatively studies the mechanism. In view of this, based on the review of previous literature, this paper summarizes and summarizes the mechanism of cultural capital affecting residents’ participation in physical activities, puts forward research hypotheses and tests them. In view of the particularity of residents’ physical activity participation, it does not involve a special issue of objective cultural capital form. Therefore, this study will focus on two dimensions: specific cultural capital and institutionalized cultural capital.

## Materials and Methods

### Study Design

The research data of this paper come from the household survey data of the China Comprehensive Social Survey (CGSS) in 2017 (Chinese general social survey, CGSS) started in 2003. It is the earliest national, comprehensive and continuous academic survey project in China. The CGSS systematically and comprehensively collects data at multiple levels of society, community, family, and individual, summarizes the trend of social change, discusses topics of great scientific and practical significance, and promotes the opening and sharing of Chinese scientific research for the country. International comparative research provides data and serves as a multidisciplinary economic and social data collection platform. At present, CGSS data have become the main data source for the study of Chinese society and are widely used in scientific research, teaching and government decision-making. The 2017 China social survey data were released to the public in October 2020. These data are one of the latest data to study China’s social issues. The valid sample of China’s comprehensive social survey data in 2017 was 12,582, including 783 variables. According to the needs of the research object, this study involves variables such as gender, age, residence, occupation and education. In this study, after deleting missing values and outliers according to the needs of the study, a total of 12,414 valid data points were entered into the analysis samples.

### Variables

#### Dependent Variable

The theme of this paper is the impact of cultural capital on physical activity participation. Therefore, physical activity was the dependent variable in this study. In the data of CGSS 2017, there were problems related to this variable. The question is “A30” (Yang et al., 2022). In the past year, do you often engage in the following activities in your spare time? Participate in physical activities. 1 = every day; 2 = several times a week; 3 = several times a month; 4 = several times a year; 5 = never; 98 = do not know; 99 = refuse to answer. To ensure the validity of the research conclusions, 98 and 99 samples were removed during the research process.

#### Independent Variable

Since China’s comprehensive social survey is a macro social survey, there are no issues related to objective cultural capital in this database. Therefore, this study takes systematic cultural capital and specific cultural capital as independent variables. Among them, systematic culture capital is reflected in the acquisition of personal academic qualifications. In the questionnaire, “a7a what is your highest education level at present” has no education; private schools and literacy classes; primary school; junior high school vocational high school; ordinary high school; secondary specialized school; technical school; college (adult higher education); college (formal higher education); undergraduate (adult higher education); undergraduate (formal higher education); graduate and above; others. In the study, we transformed educational background into continuous variables, in which primary school and below = 0; high school and below = 1; college and above = 2. Specific cultural capital is reflected in the following seven questions: “A30. Have you often engaged in the following activities in your spare time in the past year?” Watch TV or DVDs; Go out to the movies; Books/newspapers/magazines; Participation in cultural activities; For example, listening to concerts, watching performances and exhibitions; Listen to music at home; Watch sports games on the spot. Options: 1 = daily; 2 = several times a week; 3 = several times a month; 4 = several times a year or less; 5 = never; 98 = do not know; 99 = refuse to answer. A total of 98 and 99 invalid samples were excluded. There may be individual selective preference when measuring specific cultural capital with seven questions. To objectively reflect the differences in specific cultural capital, in the study, we will never participate as 0 and participate as 1 and then add the scores of seven questions to convert specific cultural capital into continuous variables.

#### Intermediary Variables

Through the previous literature review and combing, we know that personal income and self-health evaluation may be the intermediary factors of cultural capital affecting residents’ participation in physical activities. Based on this, this study adopts the question “a8a.” What was your total income last year (2016) Respondents responded with specific values. To ensure the objectivity of the data, this study adds 1 to the value and takes the logarithm for analysis. The self-health assessment adopts “A15. What do you think of your current physical health status?” The options were as follows: 1 = very unhealthy; 2 = relatively unhealthy; 3 = general; 4 = relatively healthy; 5 = very healthy; 98 = do not know; 99 = refuse to answer. Ninety-eight and 99 samples were excluded.

#### Control Variables

The control variables in this study mainly use the control variables in common studies, such as gender: female = 0, male = 1; Age was transformed into a continuous variable; Urban and rural: Rural = 0; Town = 1; Work; No work = 0, work = 1 (Table 1):

**TABLE 1:** Descriptive statistics.

Variable	Obs	Mean	Std. Dev.	Min	Max
Physical activity	12,414	1.493	1.595	0	4
Education	12,414	0.849	0.721	0	2
Specific cultural capital	12,414	2.787	1.839	0	7
Income	12,414	8.755	4.131	0	16.118
Health	12,414	3.461	1.104	1	5
Gender	12,414	0.472	0.499	0	1
Age	12,414	55.054	16.863	22	107
Urban and rural	12,414	1.361	0.48	1	2
Work	12,414	0.378	0.485	0	1

#### Reliability and Validity Test

First, the reliability and validity of the data were tested by Stata 16.0 (Table 2). Similar methods are often used in computational sociology (Jin and Vai, 2014; Jin et al., 2020; Li and Liu, 2021; Yang et al., 2022).

**TABLE 2:** Reliability test.

Frequency	Scales	Cronbach.α
12,214	9	0.79

From the results in Tables 2, 3, the sample data of the questionnaire have good reliability (α-value exceeds 0.7). Additionally, the sample results have a certain degree of authenticity (the overall value exceeds 0.7). Due to the high KMO value, which is much greater than 0.7, and the *p*-value of Bartlett’s sphere test is < 0.05, the validity of the scale is good.

**TABLE 3:** Validity test.

KMO value		0.721
Bartlett’s sphericity test	χ^2^-value	32349.346
	*P*-value	0.001

### Statistical Analyses

The dependent variable of this study was participation in physical activity. Therefore, the ordinal logit model is selected for regression analysis. To clearly show the influence of different independent variables on dependent variables, this study constructs five ordered regression models for analysis. The model is a benchmark model, including four control variables: gender, age, urban and rural areas and whether there is work. Model 2 is a sequential regression model with systematic cultural capital variables. Model 3 is a sequential regression model with specific cultural capital variables added to model 2. According to the analysis of the literature review, we take personal income and health self-assessment as intermediary variables of cultural capital affecting Chinese residents’ physical activity participation. The income variable is added to model 4 for analysis. Model 5 adds health self-assessment variables to model 4 for analysis. To understand the impact mechanism of cultural capital on residents’ participation in physical activities, the study will further explore the mediating effect of personal income and health self-assessment on residents’ participation in physical activities. The above five regression models can only explain why the self-assessment of good personal income and health plays an intermediary role in the participation of cultural capital in residents’ physical activities but cannot explain the size of the intermediary effect. Therefore, the KHB decomposition method proposed by Karlson is used to solve this problem. The KHB decomposition method is used to compare the coefficients between different models in the same sample.

## Results

The regression results of model 1 show that gender, age, and urban and rural areas have a significant impact on Chinese residents’ participation in physical activities, while whether there is work has no significant impact on residents’ participation in physical activities.

The regression results of model 2 show that systematic culture capital has a significant impact on residents’ participation in sports activities. Compared with people below primary school, people with high school education and college education are more inclined to participate in physical activities, and the frequency of participation increases with the increase in education.

The regression results of model 3 show that specific cultural capital has a significant impact on residents’ participation in physical activities. The more specific cultural capital there is, the more likely people are to participate in physical activities. The selected indicators of specific cultural capital in this study are mostly specific cultural behaviors such as reading, watching performances and watching competitions.

The regression results of model 4 show that income has a significant impact on Chinese residents’ participation in physical activities. With the increase in residents’ income, the possibility of Chinese residents participating in physical activities also increases. With the addition of income variables, the regression coefficient of cultural capital changes. Among them, the coefficient of systematic culture capital decreased from 0.91 to 0.538 in the high school group and from 1.435 to 0.6 in the college or above group, but there was no significant change in the specific cultural capital group, only from 0.437 to 0.434.

Model 5 regression results show that health self-assessment has a significant impact on Chinese residents’ physical activity participation, and residents with better health self-assessment are more likely to participate in physical activities.

The above results are shown in Table 4.

**TABLE 4:** Regression model of cultural capital and physical activity.

	Model 1	Model 2	Model 3	Model 4	Model 5
	PA	PA	PA	PA	PA
Gender	0.143*** (0.034)	0.048 (0.035)	0.096*** (0.035)	0.084** (0.036)	0.072** (0.036)
Age	-0.004*** (0.001)	0.008*** (0.001)	0.019*** (0.001)	0.018*** (0.001)	0.022*** (0.001)
	1.288*** (0.04)	0.97*** (0.042)	0.679*** (0.043)	0.671*** (0.043)	0.648*** (0.043)
Urban and rural	0.062 (0.041)	-0.085** (0.041)	-0.154*** (0.042)	-0.207*** (0.044)	-0.248*** (0.045)
0bn.Edu					
1.Edu		0.91*** (0.045)	0.138*** (0.048)	0.092*** (0.048)	0.076*** (0.048)
2.Edu		1.43*** (0.059)	0.6*** (0.065)	0.375*** (0.065)	0.228*** (0.065)
Specific cultural capital			0.437*** (0.013)	0.234*** (0.013)	0.125*** (0.013)
Income				0.017*** (0.005)	0.015*** (0.005)
1bn.Health					
2.Health					0.358*** (0.11)
3.Health					0.671*** (0.106)
4.Health					0.774*** (0.106)
5.Health					1.021*** (0.111)
Observations	12,414	12,414	12,414	12,414	12,414
Pseudo *R*^2^	0.039	0.057	0.092	0.092	0.097

Standard errors are in parentheses. ***p < 0.01, **p < 0.05, *p < 0.1.

After comparison with the KHB decomposition method, the results are shown in Table 5. First, we analyze individual annual income as an intermediary variable, and the results show that the intermediary effect of individual income on specific cultural capital is 0.106, accounting for 22.1%. The intermediary effect of personal income on systematic culture capital is 0.116, accounting for 19.9%. This shows that the intermediary effect of personal income on specific cultural capital is greater than that of systematic cultural capital. Second, after analyzing health self-assessment as an intermediary variable, the results show that the intermediary effect of health self-assessment on specific cultural capital is 0.072, accounting for 20.2%; the intermediary effect of health self-assessment on systematic culture capital is 23.1%. This shows that health self-assessment plays a role in residents’ participation in physical activities through systematic cultural capital. Third, personal income and health self-assessment are analyzed as intermediary variables at the same time. The intermediary effect of the two through specific cultural capital is 0.115, accounting for 32.2%, the intermediary effect of the two through systematic culture capitalism is 0.049, and the intermediary effect accounts for 42.6%. This shows that cultural capital largely improves residents’ economic income and health self-assessment to improve residents’ physical activity participation.

**TABLE 5:** Intermediary effect analysis of individual annual income and health self-assessment.

Mediating variable	Independent variable	Total effect	Direct effect	Mediating effect	Proportion of intermediary effect
Income	Specific cultural capital	0.478	0.372	0.106***	22.1%
	Systematic culture capital	0.584	0.468	0.116***	19.9%
Health self-assessment	Specific cultural capital	0.357	0.285	0.072***	20.2%
	Systematic culture capital	0.584	0.449	0.135***	23.1%
Income + Health self-assessment	Specific cultural capital	0.357	0.242	0.115***	32.2%
	Systematic culture capital	0.584	0.335	0.249***	42.6%

Standard errors are in parenthesis ***p < 0.01, **p < 0.05, *p < 0.1.

## Discussion

According to the connotation of Bourdieu’s cultural capital, social differences and social inequalities caused by an unfair distribution of cultural capital may lead to differences in physical activity participation (Abel, 2008). Distribution inequality in social groups This study discusses the impact and mechanism of cultural capital on residents’ physical activity participation in China through five regression models and the KHB decomposition method. Through empirical regression analysis, we can still see that there is still inequality between men and women in Chinese residents’ participation in physical activities, in which men are more inclined to participate in physical activities than women. Chinese residents will also reduce their enthusiasm to participate in physical activities with age. The difference between urban and rural areas in the dual system of Chinese society is reflected not only in economic development but also in the possibility of Chinese residents participating in physical activities. Urban residents are more inclined to participate in physical activities than rural residents. At the same time, we also found that whether Chinese residents have a job has no effect on their participation in physical activities. In terms of systematic cultural capital, systematic cultural capital represented by education can promote residents’ participation in physical activities, which also responds to previous studies: physical activities are closely related to the person’s social status, so they are closely related to his or her educational capital (Engstrm, 2008). This is because high-level education means better cognitive ability and adaptability, and then you can choose a healthy lifestyle by acquiring rich health knowledge (lingguo et al., 2014). Even in Brazil, men and highly educated people are more active in physical activities in their leisure time than their peers (Sallis et al., 2016). On the other hand, the income return promotion theory holds that better education means better professional and economic income (Li and Xuhui, 2018). This will help to improve the ability of individuals to obtain physical activity resources, purchase physical activity training, venues and other optimize the allocation of physical activity resources to promote the possibility of physical activity participation. This is positively related to the orthodoxy of cultural capital put forward by Bourdieu (Pierre., 2005). In other words, residents’ education plays an orthodox role in residents’ cultural capital, and cultural orthodoxy is just a naturalized social difference. It is not a real natural difference but a mandatory effect through hierarchical and differentiated cultural capital in the dominant relationship. In terms of specific cultural capital, the number of Chinese residents participating in recreational and sports activities affects their participation in physical activities. This is consistent with Liu’s research conclusion, that is, Chinese groups who like watching sports competitions are more inclined to participate in sports activities than those who do not like watching sports programs (Liu and Liu, 2021). Generally, there is a close relationship between residents’ personalities. Good cultural class habits do not exist in isolation. They are accumulated by residents who spend much time and energy, which explains why residents’ specific cultural capital can explain the internal logic of residents’ participation in physical activities.

In terms of the influence mechanism of cultural capital, we find that the action mechanism of systematic culture capital and specific cultural capital on physical activities may be realized through two types of intermediary variables: one is economic income, because good cultural capital means higher economic income, to obtain more physical activity resources, such as training and venues. Some studies have found that for teenagers, high family income helps them to participate in more physical exercise activities, and the types of sports involved are related to family income (Kantomaa et al., 2007). The other is self-rated health, because good cultural capital means that individuals are more likely to understand and pay attention to their own health status, to encourage residents to participate in physical activities to maintain this state. In the empirical study of this paper, Chinese residents’ health awareness and physical activity participation are in a benign state. However, it is also found that after adding the variables of health self-assessment, the coefficient of systematic culture capital decreases, which shows that health self-assessment, as systematic culture capital, plays an intermediary role in participation in physical activities. Health self-assessment also plays an intermediary role in the specific cultural capital on residents’ physical activity participation.

The impact of cultural capital on residents’ participation in physical activities can be realized by increasing residents’ income and improving their self-assessment of health. That is, (1) the higher the systematic culture capital, the more frequently they participate in physical activities, perhaps because people with higher education have more social wealth and more understanding of the importance of physical activities in Chinese society, or because physical activities have become a way to highlight the social class and there is a “separation” between social classes. (2) The higher the specific cultural capital, the more frequently the residents participate in physical activities. Specific cultural capital is generally residents’ cultural activities. Compared with cultural activities, cultural activities need certain aesthetics and sentiment. Generally, residents’ social life corresponds to their own lifestyle and track. Therefore, residents’ participation in cultural life will improve the frequency of residents’ participation in physical activities. Second, through the intermediary analysis results, we can draw the following conclusions: (1) personal income and health self-assessment play an intermediary role in promoting residents’ participation in physical activities. The increase in cultural capital will enable more people with income and the ability to pay to choose physical activities. At the same time, people with more cultural capital are more likely to participate in physical activities by improving their awareness of health. (2) Personal income and health status play an intermediary role in the promotion of physical activities by cultural capital.

## Conclusion

Therefore, the research conclusions of this paper are as follows: first, in the strategy of promoting the implementation of national physical activities, while promoting publicity in the past, we should pay more attention to the role of cultural capital in promoting residents’ physical activities and take the promotion of cultural capital as one of the ways to improve residents’ access; second, we should increase residents’ access to specific cultural capital by means of culture and art and increase the possibility of Chinese residents participating in physical activities through this mechanism; third, by increasing the number of years of education, on the one hand, we should improve residents’ understanding of the importance of physical activities, on the other hand, we should improve residents’ income ability and improve residents’ participation in physical activities.

## Data Availability Statement

The datasets presented in this study can be found in online repositories. The names of the repository/repositories and accession number(s) can be found below: http://cgss.ruc.edu.cn/.

## Author Contributions

WL drafted the work and revised it critically for important intellectual content. HR contributed to the rewriting of the manuscript. Both authors contributed to the article and approved the submitted version.

## Conflict of Interest

The authors declare that the research was conducted in the absence of any commercial or financial relationships that could be construed as a potential conflict of interest.

## Publisher’s Note

All claims expressed in this article are solely those of the authors and do not necessarily represent those of their affiliated organizations, or those of the publisher, the editors and the reviewers. Any product that may be evaluated in this article, or claim that may be made by its manufacturer, is not guaranteed or endorsed by the publisher.
